# For Genomes, Repetition Breeds Diversity

**DOI:** 10.1371/journal.pbio.1001079

**Published:** 2011-06-14

**Authors:** Richard Robinson

**Affiliations:** Freelance Science Writer, Sherborn, Massachusetts, United States of America

**Figure:**
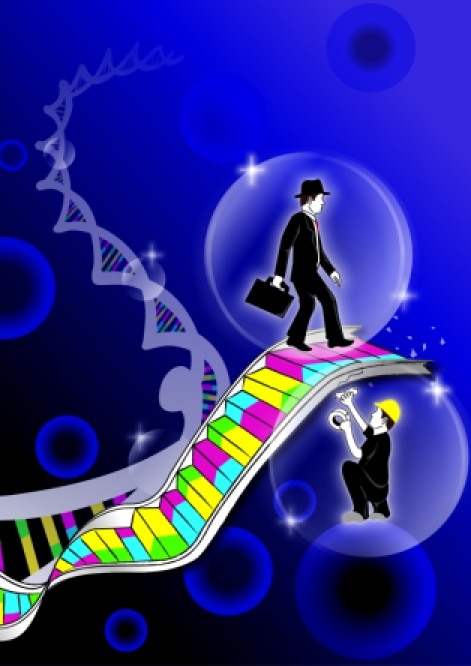
Repair on the run: recidivist, repeat-sequence induced repair processes result in the accumulation of clusters of mutations.

A long-time observation in genomes from bacteria to humans is that the level of nucleotide diversity varies from region to region within the genome. The sequence at some spots is virtually identical among all individuals in a population, while at other spots, variation abounds. What accounts for this differential variability from place to place within the genome? In this issue of *PloS Biology,* Michael McDonald, Jun-Yi Leu, and colleagues provide evidence that one prominent hypothesis doesn't explain all the facts, while another, less popular one does.

Their work concerns how insertions and deletions (“indels”) contribute to the sequence variability within a region, and the relative importance of indels to other factors. In the most widely held model, the “mutagenic indel” hypothesis, a heterozygous indel causes the DNA repair machinery to sprinkle the surrounding region with substitutions (the essence of sequence variability) in the process of attempting to correct the mismatch. A key prediction of the hypothesis is that, because the repair machinery is only called into play when sequences on homologous chromosomes differ, once the indel becomes homozygous in the population (i.e., all individuals have it on both chromosomes), there is nothing left to repair, and the accumulation of substitutions should end.

In contrast, the “regional differences” hypothesis posits that substitutions arise because of peculiarities of the local genomic environment, independent of the presence or heterozygosity of indels, and thus should continue to accumulate substitutions whether or not the indel is homozygous in the population, or even present at a particular spot.

The authors began their test of the mutagenic indel hypothesis by examining nucleotide diversity in a prokaryote, the gut bacterium *E. coli*. Prokaryotes are haploid; paired chromosomes exist for only a brief period during the life cycle, severely limiting the opportunity for diploidy-based DNA repair. Since the mutagenic indel hypothesis relies on this event, there should be little opportunity for nucleotide diversity to accumulate over time as it does in diploid eukaryotes. Thus, younger indels should have accumulated just as many substitutions around them as older ones. Instead, the authors found, older *E. coli* indels were surrounded by many more substitutions, suggesting that, despite the absence of diploidy and its associated repair mechanisms, substitutions continue to accumulate around indels over time.

A second test was to compare nucleotide diversity in regions without indels to comparable regions with indels. If indels promote substitutions (and therefore diversity), the region surrounding the indel should be more diverse, and the region without it should be no more diverse than expected from the background rate of sequence change. This comparison is trickier than it sounds, since it requires knowing ahead of time which of two similar sequences contains an indel. The authors proceeded by comparing similar regions in two different bacterial strains, and using the sequence from a third strain to infer the ancestral sequence. Contrary to the mutagenic indel hypothesis, they found that diversity in both sequences was elevated above the background, but that the sequence without the indel was just as diverse as the sequence with the indel.

Furthermore, while indels had an acute effect near the time of mutation, that effect diminished over time, while the regional effect persisted. This suggested that the sequence of the region, not the presence of the indel, was controlling the diversity level. And what was true for bacteria was also true for yeast and flies: indels caused a one-time spike in diversity, while the effect of the region was constant.

So what characteristics of a region make it prone to accumulation of diversity? Repeat sequences are well known to cause indels, as the replication machinery slips and misaligns the two strands. But the authors propose a different mechanism to account for the substitutions surrounding the repeats. The authors note that two of *E. coli*'s five DNA polymerases are prone to make errors during copying. Such polymerases are recruited when replication stalls, or when DNA breaks, events often caused by repetitive DNA sequences. Thus, apart from the indel mutations, repetitive DNA should induce multiple nearby substitutions as these polymerases take over. By searching for repeat-rich regions in multiple *E. coli* genomes, they found that diversity was highest around repeat sequences even when there were no indels nearby. They found the same pattern in multiple eukaryotic genomes, including humans. Finally, they tested their hypothesis in yeast, inserting repeats and observing an increase in substitutions in surrounding sequences.

The results here will be valuable in understanding the origin and distribution of genomic sequence diversity, a critical feature of both fundamental genomic studies and evolutionary models. Their findings also have practical applications, since diversity is the raw material for all types of gene hunting techniques.

**McDonald MJ, Wang W-C, Huang H-D, Leu J-Y (2011) Clusters of Nucleotide Substitutions and Insertion/Deletion Mutations Are Associated with Repeat Sequences. doi:10.1371/journal.pbio.1000622**

